# Selecting Near-Native Protein Structures from Predicted Decoy Sets Using Ordered Graphlet Degree Similarity

**DOI:** 10.3390/genes10020132

**Published:** 2019-02-11

**Authors:** Xu Han, Li Li, Yonggang Lu

**Affiliations:** School of Information Science and Engineering, Lanzhou University, Lanzhou 730000, China; hanxu16@lzu.edu.cn (X.H.); lili15@lzu.edu.cn (L.L.)

**Keywords:** GR_score, dynamic programming, gap penalty, near-native protein, protein structure prediction

## Abstract

Effective prediction of protein tertiary structure from sequence is an important and challenging problem in computational structural biology. Ab initio protein structure prediction is based on amino acid sequence alone, thus, it has a wide application area. With the ab initio method, a large number of candidate protein structures called decoy set can be predicted, however, it is a difficult problem to select a good near-native structure from the predicted decoy set. In this work we propose a new method for selecting the near-native structure from the decoy set based on both contact map overlap (CMO) and graphlets. By generalizing graphlets to ordered graphs, and using a dynamic programming to select the optimal alignment with an introduced gap penalty, a GR_score is defined for calculating the similarity between the three-dimensional (3D) decoy structures. The proposed method was applied to all 54 single-domain targets in CASP11 and all 43 targets in CASP10, and ensemble clustering was used to cluster the protein decoy structures based on the computed CR_scores. The most popular centroid structure was selected as the near-native structure. The experiments showed that compared to the SPICKER method, which is used in I-TASSER, the proposed method can usually select better near-native structures in terms of the similarity between the selected structure and the true native structure.

## 1. Introduction

The human genome project was first proposed by American scientists in 1985 and officially launched in 1990 [1]. Its purpose is to determine the nucleotide sequence consisting of three billion base pairs contained in a human chromosome, thereby mapping the human genome and identifying the genes and their sequences to decipher humans. With the completion of the program, the gene sequence can be obtained by measuring the obtained map, and the sequence of the corresponding protein can also be inferred using the genetic central dogma [2]. Since the function of genes can be studied via the study of the corresponding proteins produced through gene expression, the use of bioinformatics to discover the function of a protein product of a gene becomes more and more significant. In fact, determining protein functions from genomic sequences is a central goal of bioinformatics [3]. Since the function of proteins is determined by its tertiary structure, the prediction of tertiary structure based on protein sequences is a very important problem.

It is known that the number of known protein structures increases exponentially. By the end of the decade, the PDB [4] database size will be more than 150,000 structures at the current rate. However, the newly published UniProtKB/TrEMBL [5] protein database in Jan, 2019 contains 139,694,261 sequence entries. Hence, only a very small part of them have experimentally solved structures. Therefore, protein tertiary structure prediction becomes an important and challenging problem in computational structural biology.

Although many protein tertiary structure prediction methods have been proposed, there is no consensus on which one is the best [6,7]. There are usually three kind of structure prediction methods: homology modeling, threading or fold recognition, and ab initio modeling [8]. Both homology modeling and threading require known protein structures as templates, thus, they are difficult to be successfully applied in the absence of template structures. In contrast, ab initio modeling does not require a known structure: it directly predicts its spatial structure from the protein sequence. Different from these methods, which directly predict the tertiary structures, there are also methods to predict contact maps of the proteins from sequence information [9,10]. Contact maps can be predicted by finding correlated pairs of amino acids in multiple sequence alignments, or using neural network approaches. The predicted contact maps can then be used to help the tertiary structure prediction of the proteins. To help the development of high-quality protein tertiary structure prediction methods, a worldwide experiment called Critical Assessment of Protein Structure Prediction (CASP) has been held every two years since 1994 [11]. The goal of the CASP is to evaluate existing protein structure prediction methods or detect their flaws. CASP provides research groups with an opportunity to objectively test their structure prediction methods and delivers an independent assessment of the state of the art in protein structure modeling to the research community and software users. The decoy sets, generated by I-TASSER, of single-domain targets in the CASP11 [12] and CASP10 [13] were used in our experiments. These decoy sets can be downloaded from the Zhang Lab website [14].

One of the challenges in designing the ab initio structure prediction method is to select the best near-native model from a large number of predicted decoy structures. Using clustering methods based on structure similarity score have been shown to be superior to using energy function in selecting the near-native structures [15]. To use the clustering methods, a key problem is the computation of the protein structure similarity.

Many tools for comparing protein structures and computing structure similarity have been developed. One type of the comparison methods is based on the model superposition, which can be further divided into two categories: the rigid-body approaches and flexible alignment approaches. The rigid-body approaches consider the proteins as rigid objects and aim to find alignments that have the maximum number of mapped residues and the minimum deviations between the mapped structures. The rigid-body approaches mainly differ in how they combine these two objectives [16]. The final score is often expressed in terms of root mean square deviation (RMSD). Combinatorial extension (CE) [17] is a typical example of rigid structure comparison method. It aligns protein structures by chaining the consecutive aligned fragment pairs (AFPs) without twists. These AFPs are combined to evaluate the protein similarity. Global distance test (GDT) [18], also written as GDT-TS (GDT total score), is one of the scores developed to overcome shortcomings of RMSD. The GDT-TS measures the structure similarity by quantifying the number of corresponding atoms in the model that can be superposed within a set of predefined tolerance thresholds to the reference structure. Unlike RMSD, GDT-TS is more robust against small fragments movements, benefited from using several superposition thresholds. The GDT-TS is now a major assessment criterion in CASP. The template modeling score (TM-score) [19] is a variation of the Levitt-Gerstein (LG) score to assess the quality of protein structure templates and predicted full-length models. All the residues of the modeled proteins are evaluated by a protein size dependent scale, rather than using a specific distance cutoff and focusing only on the fractions of structures as in the GDT-TS. TM-score is more sensitive to the correctness of global topology than the local structural errors, while the RMSD measure is sensitive to local small disorientations which may result in a big overall RMSD change even though the core region of the model may be correct. Because proteins are flexible molecules and can undergo large conformational changes that are not captured by the rigid-body approaches, flexible alignment methods have also been developed. Flexible alignment methods overcome the limitations of the rigid body approaches by either allowing twists between rigidly aligned fragments or by only maximizing local similarities (in terms of Euclidean distance) [20]. One of the typical flexible alignment methods is FATCAT (flexible structure alignment by chaining aligned fragment pairs with twists) [20]. FATCAT is an improvement based on CE. It first identifies the local AFPs and then produces an optimal combination of these AFPs using dynamic programming, where twists and gap penalty are used to allow flexible alignments.

Another type of the protein structure comparison methods is not based on the model superposition. One of the methods is Contact Area Difference (CAD) [21], which evaluates the structure similarities based on contacts. It computes the structure similarity by measuring the differences between the physical contacts of a model and a reference structure, without supposition of the two models. The local Distance Difference Test (lDDT) [22] is another superposition free score that evaluates local distance differences of all atoms in a model, including validation of stereochemical plausibility. The reference can be a single structure, or an ensemble of equivalent structures. It is computed over all pairs of atoms in the reference structure at a distance closer than a predefined threshold, and not belonging to the same residue.

There are also methods developed specially for evaluating predicted decoys using both energy functions and the structure information. The random forest-based model quality assessment (RFMQA) [23] predicts a relative score of a decoy model by using its secondary structure, solvent accessibility and knowledge-based potential energy terms. The support-vector-machine-based single-model quality assessment (SVMQA) [24] is trained to predict TM-score and GDT_TS score based on both statistical potential energy terms and structure consistency features. 

In this article, a new protein structure similarity score, called the GR_score, was defined based on maximum Contact Map Overlap (CMO) [25] which is a superposition free protein structure alignment method defined by Godzik and Skolnick, and the ordered graphlet degree [26] which is a new systematic measure of a network’s local structure similarity. The superposition free structure alignment methods based on contact maps may capture both the local structure similarities from contact maps and the global structure similarities using dynamic programming. Using the ordered graphlet degree can further improve the measuring of the local structure similarities by comparing the local topology structures. Thus, the proposed GR_score can help in measuring the decoy structure similarities, and in selecting the near-native models from a large number of predicted decoy models in ab initio structure prediction.

## 2. Materials and Methods 

### 2.1. Maximum Contact Map Overlap (CMO)

**Figure 1:**
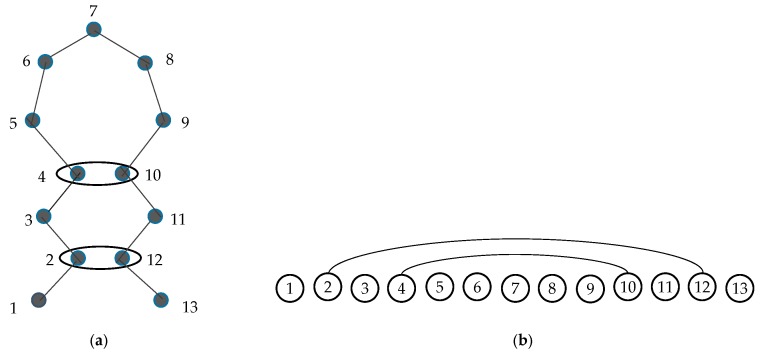
(**a**) Schematic diagram of a protein backbone. Amino acid 2 is in contact with 12 and 4 is in contact with 10 (the distance between two nodes is less than ɛ). (**b**) The corresponding contact map graph, where two edges connect node 2 with 12 and 4 with 10 [27].

A contact map is an ordered graph, CM=(V,E), where nodes *V* and edges *E* are defined as follows. Each node in *V* represents an amino acid of a protein. It leads to a strict total ordering of the nodes: for two different nodes u and w, either u< w if u is before w in the protein sequence or u> w otherwise. The two nodes u and w are connected by an edge (u,w)∈E, if and only if the Euclidean distance between the *C_α_* atoms of the corresponding amino acids is less than a given threshold *ɛ*. This is presented in Figure 1 [27].

### 2.2. Graphlets and Graphlet Degrees

**Figure 2:**
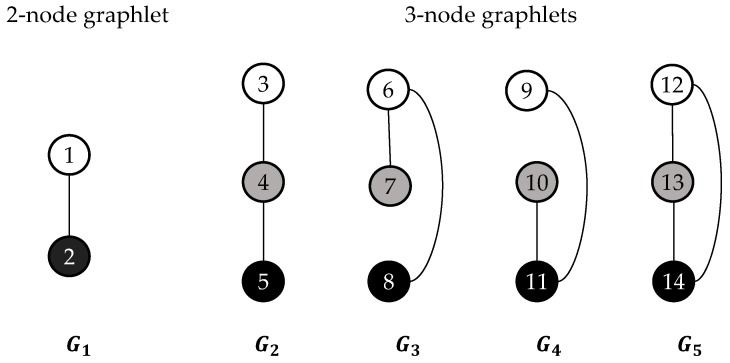
The five 2-node and 3-node ordered graphlets and the corresponding 14 automorphism orbits. The ordering of the graphlet nodes in each graphlet $G_{i},\;i\in \{1,\ldots ,5$} is represented by their colors: white nodes <gray nodes<black nodes [27].

Graphlets are small, connected, non-isomorphic and induced subgraphs of a larger graph G=(V, E) having n ≥ 2 nodes [27]. Some nodes are identical to each other topologically within each graphlet, which is considered to belong to the same automorphism orbit to represent that a graphlet can touch a node in V by different ways topologically. The concepts used to summarize the graphlets degree are: the graphlet degree of node n, represented by $d_{n}^{i}$, is the number of times a graphlet touches node n at orbit i. In the graph degree distribution protocol, the degree distribution is extended to 73 graph degree distributions by using all 2-5 nodes and their corresponding 73 automorphism orbits (the first of the 73 graph degree distributions is the degree distribution) [28]. The $i^{th}$ ordered graphlet degree of node u, represented by $d_{u,\;}^{i}$, is the number of times an ordered graphlet touches the node u at orbit i. To reduce the calculation times, the five 2-node and 3-node ordered graphlets have been chosen to define 14 orbits (see Figure 2) [27]. Therefore, a 14-dimensional vector ($d_{u,\;}^{1}d_{u}^{2},\ldots ,d_{u}^{14}$) could describe each node u of a contact map. For a given contact map CM=(V,E), there would be a limitation of the degree of a node by the number of residues that can fit in a sphere with radius ɛ. In fact, a linear worst time complexity could be led by using a distance threshold ɛ of 7.5 Å.

### 2.3. TM-Score

The TM-score [19] is intended as a more accurate measure of the protein structure similarity than RMSD and GDT-TS. It gives the residue pairs at smaller distance higher weights than those at larger distances and normalized by the length of the target proteins, thus, it can represent the global structure similarities better than RMSD or GDT-TS measures. The TM-score is between 0 and 1, where 1 indicates a perfect match between two structures. Generally, scores below 0.2 correspond to randomly chosen unrelated proteins. The score of the structures roughly having the same fold is higher than 0.5.

### 2.4. SPICKER

SPICKER is an iterative clustering method to identify near-native protein folds developed by Zhang and Jeffery [29]. The procedure of selecting protein structure by this clustering method is as follows. First, a self-adjusting cutoff between 7.5 to 12 Å is found in an iterative way to make sure that the largest cluster contains less than 70% and more than 15% of total decoys. Second, another iterated approach is applied to identify the cluster with the most neighbors under the cutoff excluding the members of cluster found in the previous iterations. Finally, an averaging model, called final model, is built from all the decoy members of the cluster in the current iteration.

### 2.5. Ensemble Clustering

Using the ensemble clustering method as introduced in [30] can avoid local optimality. The most popular centroid structure identified in the ensemble clustering is selected as the near-native structure in the proposed method. The method includes two steps: constructing a distance matrix for the decoy set using a similarity score, and finding the most possible largest cluster centroid using an ensemble k-medoids. A confidence score as described in [30] is used to select the cluster centroid with the maximum score as the near-native structure.

### 2.6. GR_score 

#### 2.6.1. Ordered Graphlet Degree Similarity.

Only *C_α_* atoms were used in the structure comparison in the proposed method. For two proteins *A* and *B*, u and w are the different *C_α_* atoms of the two proteins. Based on graphlet degrees, between two nodes u and w, the order graphlet degree similarity is defined as follows [27]:

(1) $$\;S\;\left(u,w\right)={\left(\frac{1}{14}\overset{14}{\underset{i=1}{\sum }}\frac{min\left(d_{u}^{i},\;\;\;d_{w}^{i}\right)+1}{max\left(d_{u}^{i},\;\;\;d_{w\;}^{i}\right)+1}\right)}^{2}$$

the range of the similarity score is from 0 to 1. The two nodes having similar local topologies will have a high similarity score.

#### 2.6.2. Structure Alignment Algorithm.

The alignment between two structures having, respectively, $n_{1}$ and $n_{2}$ nodes was computed using the Needleman-Wunsch dynamic programming algorithm [31] as in the original CMO, where the score of mapping two nodes is their ordered graphlet degree similarity defined in (1). It corresponds to the following dynamic programming procedure:

(2) $$\begin{array}{c} T\left[u,0\right]=0,\\ T\left[0,w\right]=0,\\ T\left[u,w\right]=max\left(\begin{array}{c} T\left[u-1\right]\left[w-1\right]+S\left[u,w\right],\\ T\left[u-1\right]\left[w\right]-g,\\ T\left[u\right]\left[w-1\right]-g \end{array}\right) \end{array}$$

where the gap penalty g is defined as follows:

(3) $$g=\alpha \times \frac{\sum_{u=1}^{n_{1}}\sum_{w=1}^{n_{2}}S\left(u,w\right)}{n_{1}n_{2}}$$

where α is a constant parameter that will be discussed in Section 3.2.

#### 2.6.3. Definition of the GR_score. 

The dynamic programming algorithm introduced in the above section produces the T[u,w] matrix, where $u\in \left[1,n_{1}\right]$ and $w\in \left[1,n_{2}\right]$. Thus, the final similarity score of the two proteins is defined as follows:

(4) $$GR\_score=\;\frac{T\left[n_{1},n_{2}\right]}{min\left(n_{1},n_{2}\right)}$$

The range of the similarity score is also from 0 to 1. The closer the value of the GR_score is to 1, the higher the similarity of the two structures; the closer the value of the GR_score is to 0, the lower the structural similarity of the two proteins.

### 2.7. Constructing the Distance Matrix

To get the distance matrix for the clustering method, a similarity matrix for the decoys needed to be constructed, and then we can get the distance matrix by defining distance = 1−similarity. The distance matrix is a symmetric matrix whose diagonal elements are all 0. The element in $l^{th}$ row and $j^{th}$ column represents the dissimilarity between two decoys l and j.

### 2.8. Select the Near-native Structure using Ensemble Clustering

K-medoids was ran *m* = 500 times, which was enough to ensure statistical stability, with random initialization. The times a decoy became the centroid of the largest cluster was counted. It was found that a reasonable value for parameter k used in k-medoids was five. Finally, to consider both the size and the internal similarity of a cluster in selecting the near-native structure, a confidence score as defined in [30] was used. The centroid with the maximum confidence score within the cluster centroids whose count was more than 70% of the maximum count was selected as the near-native structure, where the count was the times a decoy became the centroid of the largest cluster.

## 3. Results and Discussion

### 3.1. Dataset

Up to 54 decoys sets (from CASP11) [12] and 43 decoys sets (from CASP10) [13], which are single-domain targets and have experimental native structures, were downloaded from Zhang Lab website [14]. These decoy sets contain structurally non-redundant set of protein structures from the raw decoy sets. The native structure, the generated model by SPICKER used in I-TASSER [32] sever, and the best TM-score for the target in the decoy set were also downloaded from the Zhang Lab website [14] (Supplementary materials).

### 3.2. Parameter Selection

In the dynamic programming, to select a good parameter α, four values of α, 0.2, 0.5, 1, and 2, were compared. For each decoy set, the similarity matrix was obtained by using the proposed GR_score in Section 2.6.3 using each α value. Then, the most popular centroid structure was selected as the near-native structure by the proposed method. The near-native structures selected by the proposed method and the corresponding native structures were compared using the TM-score. 

**Figure 3:**
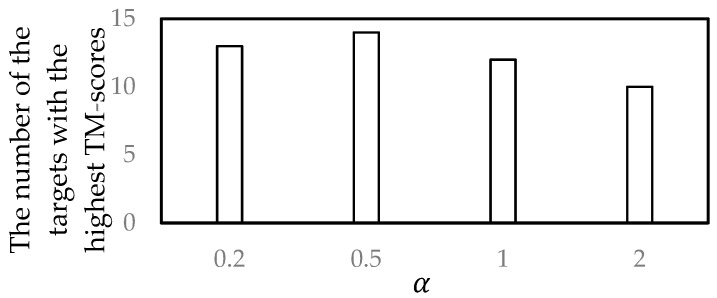
Parameter selection.

In the experiments, 54 targets from CASP11 were used. For each target, four different TM-scores were produced from four α values, and the α value that produced the highest TM-score was recorded. Finally, for each α value, the number of the targets for which the highest TM-scores were produced using the α value was counted. The numbers of the targets with the highest TM-scores for four α values are shown in Figure 3.

It can be seen from Figure 3 that when α=0.5*,* the selected near-native structures were more similar to the corresponding native structure, compared to the other α values. Thus, the parameter α was set to 0.5 in the proposed method.

### 3.3. Experimental Results

#### 3.3.1. The Experimental Results for Datasets from CASP10.

**Figure 4:**
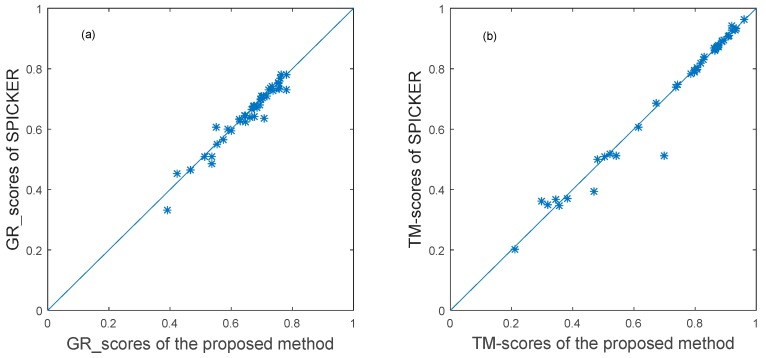
The plot of GR_scores and TM-scores produced by two methods for datasets from CASP10.

For the proposed method, the GR_score was used to calculate the similarity matrix of the 43 decoy sets from CASP10. Then, the ensemble clustering was used to select the near native structures for each target. The near-native structure selected by the proposed method and the near-native structure generated by the SPICKER method used in I-TASSER sever were compared. The TM-sore and the GR_score between the selected near-native structures and the native structure were computed. The results are shown in the scatter plots in Figure 4, in which each target protein is represented as one point. The *x*-axis represents the GR_score or TM-score produced by the proposed method, and the *y*-axis represents the scores produced by the SPICKER method for the same target. The blue diagonal line in Figure 4 represents y=x. The same score does not necessarily mean the same model.

The details of the comparison can also be found in Table 1, in which the first column is the ID of the target protein, the second column and the third column are the GR_scores of the selected near-native models by the proposed method and the SPICKER method, the fourth column and the fifth column are the TM-scores of the selected near-native model by the proposed method and the SPICKER method. All the scores were computed between the selected near-native model and the corresponding native structure.

**Figure 5:**
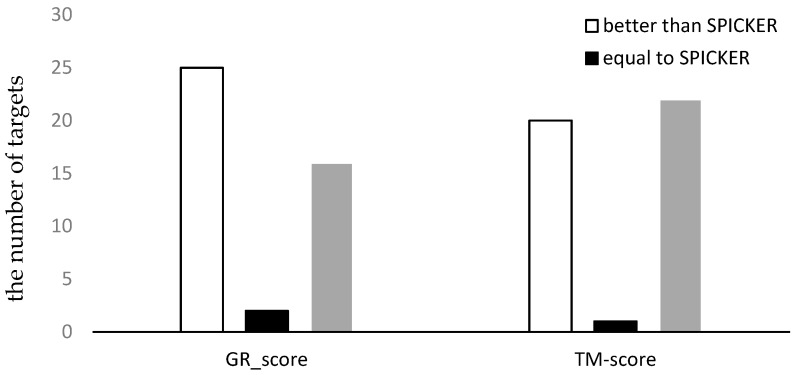
The comparison of the two methods using both GR_score and TM-score for datasets from CASP10.

To better understand the results, the number of the targets for which each method produced the better results was counted. The results are shown in Figure 5, where the white bar represents the number of decoy sets for which our method produces better results than SPICKER, the gray bar represents the number of decoy sets for which our method produces worse results than SPICKER, and the black bar represents the number of the similar results produced by the two methods. It can be seen from the left part of Figure 5 that the proposed method selected more near-native structures with higher GR_scores, compared to the SPICKER method. However, when measuring the similarity using the TM-score, the SPICKER method produced more near-native structures with higher scores, as can be seen from the right part of Figure 5, although the difference was smaller compared to the GR_score result on the left part of Figure 5. This may be due to fact that the similarity measure used in the proposed method is GR_score, instead of the TM-score.

#### 3.3.2. The Experimental Results for Datasets from CASP11.

**Figure 6:**
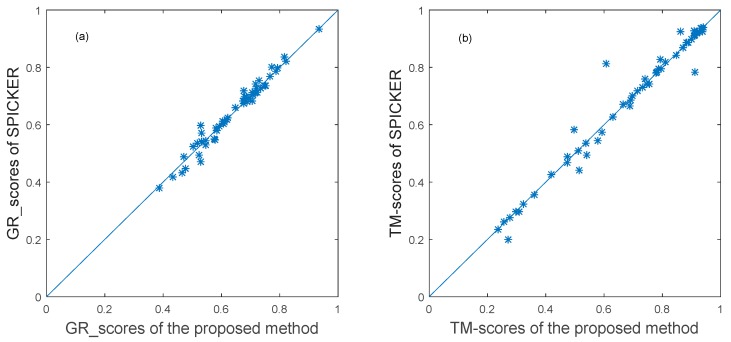
The plot of GR_scores and TM-scores produced by two methods for datasets from CASP11.

To further evaluate the proposed method, it was also applied to the 54 decoy sets from CASP11. The near-native structure selected by the proposed method and the near-native structure generated by the SPICKER method used in I-TASSER sever were compared. The results of the GR_score are shown in the left scatter plot in Figure 6, while the results of the TM-score are shown in the left scatter plot in Figure 6.

Detailed results with scores for all the targets are shown in Table 2. 

**Figure 7:**
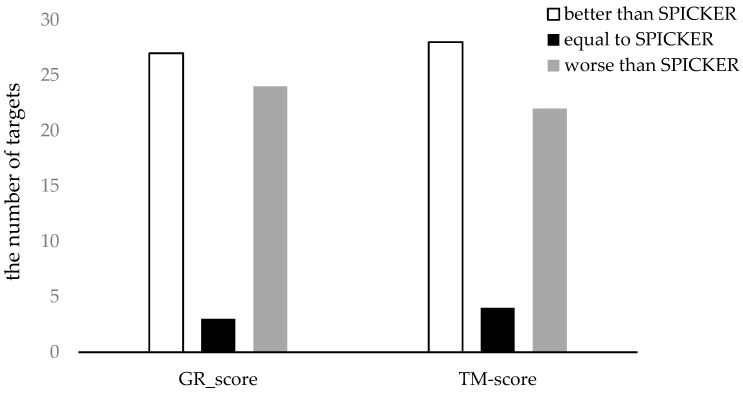
The comparison of the two methods using both GR_score and TM-score for datasets from CASP11.

To clearly represent the results, the number of the targets for which each method produces the better results was counted. The results are shown in Figure 7. It can be seen from the Figure 7 that the proposed method can select better near-native structures for more targets compared to the SPICKER method, evaluated either with GR_scores or with TM-scores.

**Figure 8:**
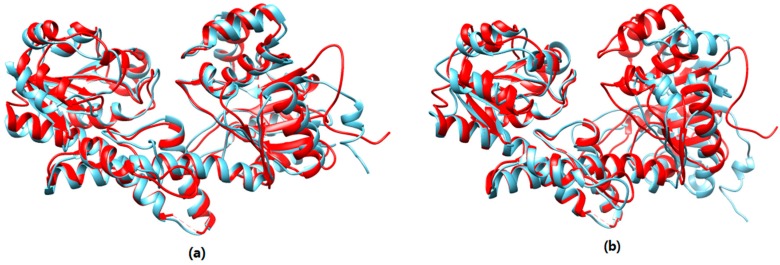
(**a**) The superposition of T0851 native structure and the near-native structure selected by the proposed method. (**b**) The super-position of T0851 native structure and the model selected by SPICKER.

Taking target T0851 as an example, Figure 8 shows the superposition between the native structure and the near-native structure found by the proposed method and the near-native structure selected by SPICKER. The red model is the native structure and the blue is the structure selected by the proposed method in Figure 8a, the other blue structure is generated by SPICKER in Figure 8b. It can be seen from Figure 8 that the SPICKER model has an obvious mismatch in the right half part of the protein.

## 4. Conclusions

In this paper, we have proposed a new similarity score, GR_score, for comparing two protein structures based on both CMO and order graphlet degrees. The introduced GR_score can serve as a new assessment criterion for protein structure comparison. It is shown that the proposed GR_score along with the ensemble clustering can be used to select the near-native structures from the decoy sets. Compared to the state-of-the-art SPICKER method, the proposed method can select more high quality near-native structures if evaluated using the GR_score for datasets from both CASP10 and CASP11. In future work, we will continue to improve the computation of the similarity scores between protein structures, and to evaluate the similarity scores from more aspects.

## Figures and Tables

**Table 1:** The Comparison of GR_scores and TM-scores for datasets from CASP10. The bold number indicates the highest GR_score or TM-score for each target.

Target ID	GR_Scores of the Proposed Method	GR_Scores of SPICKER	TM-Scores of the Proposed Method	TM-Scores of SPICKER
T0644	0.764	**0.781**	**0.869**	0.865
T0645	**0.666**	0.664	**0.932**	0.929
T0649	0.423	**0.454**	**0.382**	0.369
T0650	0.702	**0.703**	0.876	**0.877**
T0654	0.626	**0.634**	0.819	**0.820**
T0655	0.672	**0.677**	0.743	**0.749**
T0657	**0.693**	0.681	0.827	**0.831**
T0659	0.753	**0.754**	**0.909**	0.906
T0662	0.727	**0.737**	**0.798**	0.796
T0664	**0.684**	0.671	**0.936**	0.934
T0665	**0.756**	0.732	0.738	**0.739**
T0667	0.643	**0.646**	**0.807**	0.803
T0669	**0.675**	0.641	**0.614**	0.606
T0672	0.590	**0.601**	**0.785**	0.784
T0673	**0.535**	0.509	0.317	**0.350**
T0675	0.552	**0.606**	**0.356**	0.346
T0676	**0.553**	0.505	0.503	**0.510**
T0678	**0.599**	0.594	0.297	**0.362**
T0679	**0.648**	0.625	**0.807**	0.798
T0680	**0.709**	0.637	**0.699**	0.513
T0681	0.700	**0.710**	**0.875**	0.872
T0683	**0.660**	0.639	0.888	**0.889**
T0688	**0.629**	0.627	0.862	**0.869**
T0689	0.734	**0.742**	0.919	**0.927**
T0691	**0.468**	0.464	0.480	**0.500**
T0692	0.704	**0.710**	0.921	**0.942**
T0703	**0.673**	**0.673**	0.894	**0.895**
T0704	0.675	0.677	0.831	**0.838**
T0708	**0.736**	0.726	0.887	**0.891**
T0714	**0.781**	**0.781**	**0.911**	**0.911**
T0716	**0.753**	0.752	0.674	**0.685**
T0721	**0.716**	0.710	0.870	**0.872**
T0722	**0.780**	0.729	**0.541**	0.513
T0723	0.697	**0.702**	**0.866**	0.859
T0733	**0.647**	0.645	**0.864**	0.863
T0749	**0.755**	0.737	0.961	**0.963**
T0752	0.721	**0.729**	0.873	**0.874**
T0753	0.696	**0.698**	**0.797**	0.790
T0757	0.760	**0.768**	0.888	**0.893**
R0001	**0.390**	0.333	**0.212**	0.202
R0008	**0.574**	0.566	**0.522**	0.519
R0014	**0.536**	0.486	**0.469**	0.393
R0018	**0.514**	0.508	0.345	**0.366**
Average	0.657	0.651	0.729	0.726

**Table 2:** The Comparison of GR_scores and TM-scores for datasets from CASP11. The bold number indicates the highest GR_score or TM-score for each target.

Target ID	GR_Scores of the Proposed Method	GR_Scores of SPICKER	TM-Scores of the Proposed Method	TM-Scores of SPICKER
T0759	**0.547**	0.530	**0.362**	0.356
T0762	0.721	**0.728**	0.921	**0.925**
T0763	**0.432**	0.416	**0.272**	0.198
T0764	0.679	**0.697**	0.883	**0.885**
T0765	0.530	**0.597**	0.740	**0.761**
T0766	0.772	**0.800**	**0.938**	0.935
**T0768**	**0.547**	**0.544**	**0.629**	**0.626**
T0769	**0.707**	0.684	**0.747**	0.741
T0773	0.729	**0.754**	0.608	**0.812**
T0778	0.817	**0.836**	0.910	**0.929**
T0782	0.580	**0.589**	**0.691**	0.687
T0784	0.717	**0.742**	0.932	**0.937**
T0785	**0.387**	0.380	0.257	**0.261**
T0786	**0.618**	**0.618**	**0.782**	**0.782**
T0787	**0.594**	0.593	**0.235**	**0.235**
T0788	**0.688**	0.681	**0.901**	0.897
T0792	**0.750**	0.735	0.665	**0.672**
T0796	**0.585**	0.579	**0.687**	0.666
T0797	**0.934**	**0.934**	0.794	**0.826**
T0798	**0.823**	0.822	0.936	**0.937**
T0800	**0.523**	0.495	**0.592**	0.575
T0801	**0.710**	0.703	**0.937**	0.926
T0803	**0.464**	0.431	**0.475**	0.467
T0805	0.706	**0.713**	**0.848**	0.843
T0807	**0.693**	0.691	0.911	**0.913**
T0811	**0.736**	0.727	**0.942**	0.941
T0812	0.503	**0.525**	**0.539**	0.536
T0813	**0.724**	0.712	0.921	**0.922**
T0815	0.794	**0.798**	**0.888**	0.885
T0816	0.647	**0.658**	0.298	0.296
T0817	**0.678**	0.675	0.715	**0.718**
T0819	0.685	**0.699**	0.916	**0.920**
T0820	0.472	**0.488**	**0.325**	0.324
T0821	0.768	**0.769**	0.810	**0.818**
T0822	**0.528**	0.470	**0.514**	0.442
T0823	0.621	**0.623**	0.778	**0.779**
T0824	**0.477**	0.446	**0.308**	0.296
T0825	**0.786**	0.785	**0.511**	0.509
T0829	0.603	**0.611**	0.496	**0.584**
T0833	**0.753**	0.736	**0.754**	0.743
T0835	0.531	**0.541**	0.697	**0.700**
T0836	0.532	**0.570**	**0.276**	**0.276**
T0837	**0.608**	0.604	0.418	**0.427**
T0838	**0.579**	0.548	**0.577**	0.543
T0841	**0.715**	**0.715**	0.861	**0.926**
T0843	**0.718**	0.713	**0.926**	0.924
T0847	0.673	**0.683**	**0.788**	**0.788**
T0849	**0.610**	0.608	**0.731**	0.730
T0851	0.678	**0.717**	**0.913**	0.782
T0854	0.679	**0.684**	**0.795**	0.794
T0855	**0.576**	0.551	**0.541**	0.494
T0856	0.677	**0.683**	**0.870**	0.869
T0857	0.516	**0.534**	0.475	**0.487**
T0858	0.673	**0.683**	0.908	**0.910**
Average	0.644	0.645	0.688	0.688

## Supplementary Materials

Following are available online at http://www.mdpi.com/2073-4425/10/2/132/s1, code and data used.

## References

[1] Collins F.S., Michael M., Aristides P. (2003). The human genome project: Lessons from large-scale biology. Science.

[2] Crick F. (1970). Central dogma of molecular biology. Nature.

[3] Pellegrini M., Marcotte E.M., Thompson M.J., Eisenberg D., Yeates T.O. (1999). Assigning protein functions by comparative genome analysis: protein phylogenetic profiles. Proc. Natl. Acad. Sci. USA.

[4] Berman H.M., Westbrook J., Feng Z., Gilliland G., Bhat T.N., Weissig H., Shindyalov I.N., Bourne P.E. (2000). The protein data bank. Nucl. Acids Res..

[5] UniProtKB/TrEMBL Protein database release statisics. http://www.ebi.ac.uk/uniprot/TrEMBLstats.

[6] Zhang Z. (2002). An overview of protein structure prediction: From homology to ab initio. Bioc218.

[7] Hasegawa H., Holm L. (2009). Advances and pitfalls of protein structural alignment. Curr. Opin. Struct. Biol..

[8] Yang Z., Jeffrey S. (2004). Automated structure prediction of weakly homologous proteins on a genomic scale. Proc. Natl. Acad. Sci. USA.

[9] Wang S., Sun S., Li Z., Zhang R., Xu J. (2017). Accurate de novo prediction of protein contact map by ultra-deep learning model. PLoS Comp. Biol..

[10] Hamilton N., Burrage K., Ragan M.A., Huber T. (2004). Protein contact prediction using patterns of correlation. Proteins.

[11] Moult J., Pedersen J.T., Judson R., Fidelis K. (1995). A large-scale experiment to assess protein structure prediction methods. Proteins.

[12] The 11th critical assessment of techniques for protein structure prediction. http://predictioncenter.org/casp11.

[13] The 10th critical assessment of techniques for protein structure prediction. http://predictioncenter.org/casp10.

[14] The Yang Zhang Lab. https://zhanglab.ccmb.med.umich.edu/decoys/.

[15] Shortle D., Simons K.T., Baker D. (1998). Clustering of low-energy conformations near the native structures of small proteins. Proc. Natl. Acad. Sci. USA.

[16] Godzik A. (2010). The structural alignment between two proteins: Is there a unique answer?. Protein Sci..

[17] Shindyalov I.N., Bourne P.E. (1998). Protein structure alignment by incremental combinatorial extension (CE) of the optimal path. Protein Eng..

[18] Zemla A., Venclovas C., Moult J., Fidelis K. (2015). Processing and analysis of CASP3 protein structure predictions. Proteins.

[19] Zhang Y., Skolnick J. (2004). Scoring function for automated assessment of protein structure template quality. Proteins.

[20] Ye Y., Godzik A. (2003). Flexible structure alignment by chaining aligned fragment pairs allowing twists. Bioinformatics.

[21] Kliment O., Eleonora K., Ceslovas V. (2013). CAD-score: A new contact area difference-based function for evaluation of protein structural models. Proteins.

[22] Valerio M., Marco B., Alessandro B., Torsten S. (2013). IDDT: A local superposition-free score for comparing protein structures and models using distance difference tests. Bioinformatics.

[23] Manavalan B., Lee J., Lee J. (2014). Random forest-based protein model quality assessment (RFMQA) using structural features and potential energy terms. PLoS ONE.

[24] Manavalan B., Lee J. (2017). SVMQA: Support-vector-machine-based protein single-model quality assessment. Bioinformatics.

[25] Godzik A., Skolnick J. (1994). Flexible algorithm for direct multiple alignment of protein structures and sequences. Bioinformatics.

[26] Przulj N., Corneil D.G., Jurisica I. (2004). Modeling interactome: Scale-free or geometric?. Bioinformatics.

[27] Malod-Dognin N., Przulj N. (2014). GR-Align: Fast and flexible alignment of protein 3D structures using graphlet degree similarity. Bioinformatics.

[28] Murzin A.G., Brenner S.E., Hubbard T., Chothia C. (1995). SCOP: A structural classification of proteins database for the investigation of sequences and structures. J. Mol. Biol..

[29] Zhang Y., Skolnick J. (2004). SPICKER: A clustering approach to identify near-native protein folds. J. Comput. Chem..

[30] Li L., Lu Y., Yan H. (2018). Selecting near-native protein structures from ab initio models using ensemble clustering. Quant. Biol..

[31] Needleman S.B., Wunsch C.D. (1970). A general method applicable to the search for similarities in the amino acid sequence of two proteins. J. Mol. Biol..

[32] Yang J.Y., Yan R.X., Roy A., Xu D., Poisson J., Zhang Y. (2015). The I-TASSER Suite: Protein structure and function prediction. Nat. Methods.

